# Supplementary material for: Broad protective vaccination against systemic Escherichia coli with autotransporter antigens
Source: PLoS Pathog. 2023 Feb 17;19(2):e1011082. doi: 10.1371/journal.ppat.1011082 (PMC9937491; doi:10.1371/journal.ppat.1011082)
Supplement: S1 Fig — Purified protein bands were resolved and digested in gel. The tryptic peptides were analyzed on nanospray LC-MS (liquid chromatography-mass spectrometry) system. The eluted peptides were directly electro-sprayed into mass spectrometer and analyzed by data-dependent acquisition (DDA). The coverage (the percentage of the protein sequence by identified peptides) of GST-SinH-3 was shown in (A), and the coverage of GST-SinH-123 was shown in (B). (DOCX) [file ppat.1011082.s001.docx]

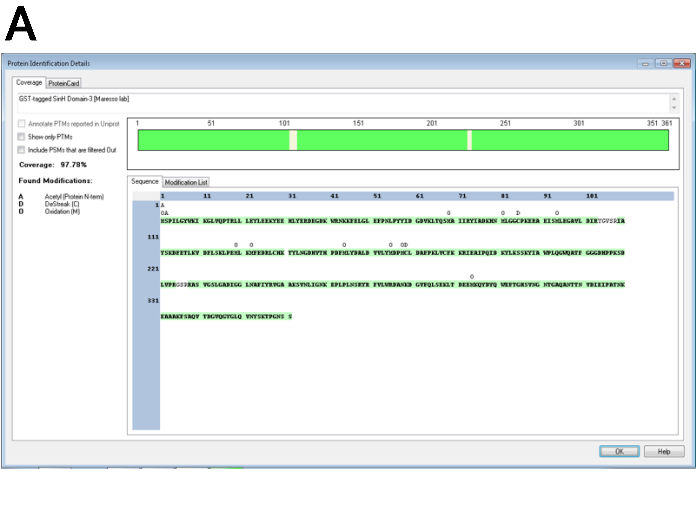

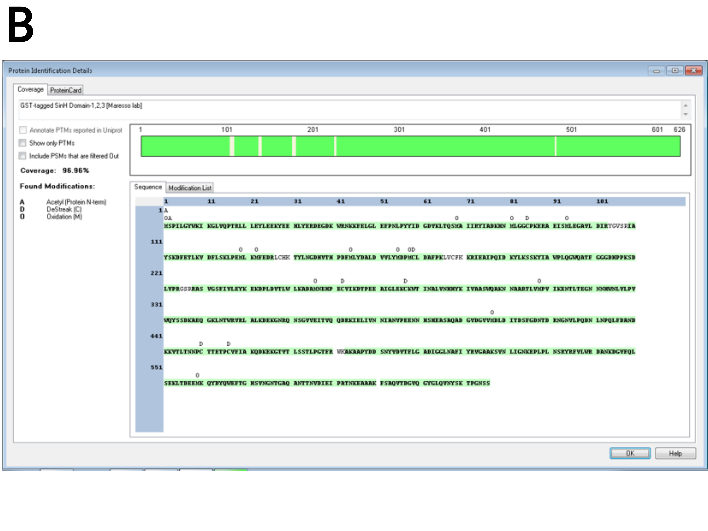


**S1 Fig. Determination of the putative GST-SinH-3 and GST-SinH-123 proteins by Mass Spectrometry per-band sequencing.** Purified protein bands were resolved and digested in gel. The tryptic peptides were analyzed on nanospray LC-MS (liquid chromatography-mass spectrometry) system. The eluted peptides were directly electro-sprayed into mass spectrometer and analyzed by data-dependent acquisition (DDA). The coverage (the percentage of the protein sequence by identified peptides) of GST-SinH-3 was shown in (A), and the coverage of GST-SinH-123 was shown in (B).
