# Supplementary material for: Broad protective vaccination against systemic Escherichia coli with autotransporter antigens
Source: PLoS Pathog. 2023 Feb 17;19(2):e1011082. doi: 10.1371/journal.ppat.1011082 (PMC9937491; doi:10.1371/journal.ppat.1011082)
Supplement: Attachment — Submitted filename: Responses to Reviewers - Final Version.docx [file ppat.1011082.s002.docx]

**Anthony William Maresso, Ph.D.**

Joseph Melnick Endowed Chair and Professor

One Baylor Plaza, BCMC 203A

MS: BCM280

HOUSTON, TEXAS 77030-3411

832-446-7284

[maresso@bcm.edu](mailto:maresso@bcm.edu)

[TAILΦR Labs](https://www.bcm.edu/research/labs-and-centers/research-centers/tailor)

Twitter [@BCMTailorLabs](https://twitter.com/BCMTailorLabs?s=20)

September, 28^th^, 2022

Kimberly Kline

Christoph Tang

Kasturi Haldar

Michael Malim

Editors

*PLOS Pathogens*

**RE:** Submission of a Revised manuscript titled, “*Broad Protective Vaccination against Systemic Escherichia coli with Autotransporter Antigens”*.

Dear Managing Editor,

Thank you for serving as the Managing Editor for our manuscript. We are encouraged that the original submission was well received and are submitting now an appropriately revised manuscript that addresses all of the reviewer’s comments. Their critiques helped improve the overall quality of the study and revised manuscript.

The revised manuscript now includes the following major changes:

1. New experimentation addressing the role of LPS in contribution to vaccine protection.
2. New experimentation including an alum-only control vaccination cohort.
3. New experimentation showing survival data from a challenge with ST131 ExPEC.
4. New experimentation showing antigen purity by SDS-PAGE.
5. Mass Spectrometry data confirming the identity of antigens.
6. Improved resolution of some figures.
7. Clarity on some statistical analyses and infection replicate numbers.
8. Added missed citations.
9. Analysis and clarity on SinH gene homologues in ST131 sequence types.
10. Elimination of typos and rephrasing or clarity of some statements.

A detailed description of these changes can be found in the document labeled “Response to Reviewers.”

We hope the revised manuscript is now suitable for publication in PloS Pathogens and thank you again for review.

Anthony William Maresso, Ph.D.

Melnick Endowed Chair and Professor

Baylor College of Medicine

**RESPONSE TO REVIEWERS**

The authors wish to thank the reviewers for their time in reviewing this study, which helped improve the manuscript. A point-by-point response to the reviewer’s comments are indicated below, as are any changes that were made to the revised manuscript (indicated by line numbers).

**Reviewer #1 (Comments for the Author):**

1. **The abstract (line 28) and results (line 121) call sinH an uncharacterized gene. However, when searching PubMed for “sinH and *E. coli*”, four references are identified. One is the genomic sequence screens described previously by the senior author’s group.**

**The most recent publication, however, has characterized the sinH gene and its respective predicted protein domains based on protein prediction software. According to the abstract, the gene was mutated and investigated in the murine urinary tract infection model. The abstract also states that a sinH mutant has a fitness defect and its gene is upregulated during urinary tract infections in women.**

**This is a 2022 paper and was likely missed during manuscript preparation, but appears to provide important background that should now be included. Shea AE et al. Loss of an Intimin-Like Protein Encoded on a Uropathogenic *E. coli* Pathogenicity Island Reduces Inflammation and Affects Interactions with the Urothelium. Infect Immun. 2022. 90:e0027521.**

**Author Response:** Thank you for pointing out these previous studies. We have taken the reviewer’s points seriously and made the following changes:

1. Of four papers mentioned above, three are now referenced in some capacity in this manuscript. The fourth search result has nothing to do with SinH in *E. coli*. The new citations include the above-mentioned paper as reference [69] and more discussion of *sinH* based on the recently published papers (lines 144 – 146).
2. We also changed some wording in the MS to update our descriptions. For example, we changed (line 47-48) from “an uncharacterized gene” to “a gene encoding an invasin-like protein.” A sentence in the introduction (lines 138-139) has been changed from “a gene encoding for an uncharacterized autotransporter protein” to now read “a gene encoding for a structural invasin-like autotransporter protein [69].” We also corrected “the generally unexplored function” to “the critical function of this gene for bladder colonization and *E. coli* infection (lines 147-148).” As the reviewer mentions, this main paper was missed because it was new.
2. **Citations are somewhat spotty. For example, when reviewing antigens that have been tried as vaccine for ExPEC, perhaps the most promising one, FimH of type 1 fimbria is not mentioned. Siderophores were also tried.**

**Author Response:** Thank you. We did make an attempt to reference those regarding FimH but mistakenly omitted the iron siderophore work. So, we made the following changes. In the sentence in the introduction about the FimH of type 1 fimbriae we changed it to read from “fimbrial adhesins” to “FimH adhesin (Type I fimbriae adhesin) (line 117 – 118).” We also added the citation concerning the siderophores and FdeC antigens which was a very important finding (lines 119 – 120). Finally, we added a few more supplementary citations in this section (lines 116 – 120).

**Other references do not always cite the primary literature. Sometimes reviews are not the most relevant articles to cite. Some are omitted altogether. A more thorough assembly of these references should be added.**

**Author Response:** Thank you, we agree. To improve upon this point, we added 33 more citations (references 66-99) which we hope is now viewed as being more comprehensive. We also modified some old references, such as references 25, 28, 59, and 60, to ensure to cite the most relevant primary literature.

**Also (beginning line 135), primary references should be provided for published strains.**

**Author Response:** Good point. We have added the primary references for the published strains (line 158-160, [95]-[97]).

**Also, unless the authors developed the three murine models in this study, primary references should be given for those models in the Methods sections. As well, the vaccination protocol should be referenced unless developed by the authors.**

**Author Response:** The three murine models and vaccination protocol were modified from the protocols or papers from our group or co-author’s groups. We have added the primary references to the vaccination (line 259, [80]), murine model of bacteremia (line 267, [26]), murine model of acute UTI (line 285, [66]), and murine model of GI tract (line 301, [26,27]).

3. **Some figures are illegible or uninterpretable as downloaded. For example Fig 1 B and C. and Fig 4A.**

**Author Response:** Thank you. The accession numbers in Fig 1B and 1C have been removed as they do not provide much additional value to the figure. The font for all three figures has been increased in size.

Additionally, we noticed that the identity histogram for figure 4A was not the identity histogram for that alignment. We have corrected this in the updated manuscript.

4. **With respect to the “purified antigens”, one can see the predicted induced bands and when cut out and subjected to mass spec, they are clearly correct, but there are many many other bands in the a-little-hard-to-see Fig 2F (perhaps incompletely destained). Presumably this is what was used for vaccination. It cannot be ruled out that these other bands may be what is affording protection and to what the ELISA is reacting.**

**The authors should show an SDS-PAGE of what was used for vaccination or a Western blot showing reactivity to only the SinH domains.**

**Author Response:** We followed up on this and have revised the MS to include a gel of the actual antigen preparations used in the study **(Data shown below)**.

In the new gel, we see there are three strong protein bands, one at about 40 kDa, one at about 70 kDa, and one at about 26 kDa. We identified these bands by mass spectrometry. For the GST-SinH-3, the protein band around 40kDa is the GST-SinH-3. Likewise, for the GST-SinH-123, the protein band around 70 kDa is the GST-SinH-123. The cover maps of the MS results are shown below.

Because the plasmid we used for both GST-SinH-3 and GST-SinH-123 was pGEX-2TK, which generates an N-terminal GST-tagged protein upon expression, and both proteins are expected to be GST-tagged proteins. The protein band around 26 kDa under both GST-SinH-3 and GST-SinH-123 bands was determined to be free GST, likely a cleavage product because of the proteolytic recognition sequence between GST and SinH. The spectra for this are shown below.

5. **Furthermore, a vaccination trial that has only alum, only sinH and the combo (the latter done) should be done.**

**Author Response:** We agree this is a good suggestion and as such performed the experiment in question. The details are presented below. The Alum-only group (30 μl/mouse, N=8) was the control group. The SinH-3 (50 μg antigen mixed with 30 μl Alum adjuvant, N=12) and the SinH-123 (50 μg antigen mixed with 30 μl Alum adjuvant, N=12) were the experimental groups. Six-week-old female BALB/cJ mice were given three subcutaneous injections of Alum-only or SinH-antigens on days 0, 14, and 28.

ExPEC sequence type 131 (ST131) strain JJ2050 were grown under the indicated conditions as described in the manuscript. Mice were injected intraperitoneally with 50 μl of the JJ2050 strain suspension (5 × 10^7^ CFU) on day 42. Mice were monitored twice a day for 10 days. Murine survival was followed with time, and moribund animals were euthanized/necropsied to determine bacterial levels in the kidneys, spleen, and liver. The organs were homogenized, and the JJ2050 bacterial load in the infected organs was quantified to determine CFU. The survival rate curve was determined using the Gehan-Breslow-Wilcoxon comparison. Significance was determined by the Kruskal-Wallis analysis of variance (ANOVA) with Dunn’s multiple comparisons correction. Symbols represent data of individual mice. One star (*) *P* < 0.05, two stars (**) *P* < 0.01, three stars (***) *P* < 0.001, four stars (****) *P* < 0.0001.

The vaccination schema used in this experiment is shown below.

All the Alum-only vaccinated control group mice died one day-post-infection (0% survival). In contrast, the mice immunized with the SinH antigens showed a 33% survival rate in the 10-day challenge period, which was statistically significant (Adjusted *P* value, SinH-3, *P*=0.0037; SinH-123, *P*=0.0090). (Alum group: 8/8 mice died in 1 d.p.i; SinH-3 group: 4/12 mice died in 1 d.p.i, 4/8 mice died in 2 d.p.i, and 4/4 mice survived until the end of 10 days; SinH-123 group: 5/12 mice died in 1 d.p.i, 3/7 mice died in 2 d.p.i, and 4/4 mice survived until the end of 10 days) **(data shown below)**.

The bacterial levels in SinH vaccinated animals showed results consistent with the survival data. Combining the counts from all organs, compared to the mice vaccinated with alum-only, mice vaccinated with SinH-3 significantly reduced the bacterial loads of JJ2050 in organs after 10 d.p.i (Adjusted *P* value, *P*<0.0001). Likewise, compared to the mice vaccinated with alum-only, the mice vaccinated with SinH-123 significantly reduced the bacterial burden of JJ2050 in organs after 2 d.p.i and 10 d.p.i (Adjusted *P* value, *P*=0.0204, *P*<0.0001). Compared to the mice that received alum-only, SinH-3 vaccinated mice had a 3.8-log reduction and SinH-123 vaccinated mice had a 6.38-log reduction in the median level of JJ2050 strain after 10 d.p.i. **(data shown below).**

These results indicate that the alum-only vaccinated group did not provide any protection against ST131 *E. coli* JJ2050 mortality or reduction in bacterial levels, whereas both SinH antigens increased survival rate with substantial reductions (4-6 log) in bacterial levels 10 d.p.i. Taken together these data suggest alum is not responsible for the observed protection for the SinH antigens. Of importance as well, this is now the third ST type that this vaccine affords some level of protection against (the other two being ST95 and ST73 as demonstrated in the original manuscript).

6. **Challenging with a sinH mutant of the ST131 strain should demonstrate no protection since it lacks the key antigen. This represents a key experiment to clarify the protection data and to demonstrate SinH domains are actually protective and not contaminants.**

**Author Response:** We think this experiment would be more nuanced than the reviewer appreciates. As indicated in the now multiple SDS PAGE gels, the mass spec analysis identified the antigens as correct, there are now 7 separate vaccine studies showing efficacy in three distinct models of infection, there is a specific double isotype antibody response to the antigens used in this study (IgG and IgA), and many log reduction in bacterial levels with now the alum only control showing consistent results. Thus, we believe it is absolutely a reasonable interpretation that SinH is providing protection against ExPEC infection in these studies. Furthermore, and perhaps more problematic, any *sinH* null strain is likely to be (given the new results herein) substantially reduced in its ability to cause disease, thereby complicating the teasing out of virulence related to suppression by the immune response and reduced attenuation.

**Reviewer #1 (Other comments):**

1. **In Fig 4E, vaccinated with SinH-3 and challenged with ST73, there are six data points (>25% of mice) that are nearly higher than any control value. Please check the statistics on this. The stats would likely take a big hit with these values**

**Author Response:** Thank you for your comment. We do not know exactly why this may be the case. In the murine model of bacteremia using ST73 and ST95, after being challenged with 5 × 10^7^ CFU ST95 or ST73 on day 42, 2/8 (ST73) and 1/7 (ST95) of SinH-3 vaccinated mice died. It could be that the deaths in these animals prior to us being able to sacrifice them caused outliers in CFU counts (higher than normal if the deceased animals sat for a period of time). To re-check the analysis here, we compared the bacterial loads (CFU) data of the control group mice with the ST73 or ST95 bacterial loads (CFU) data at 5 d.p.i of the surviving mice only. According to the results, combining the counts from all organs, mice vaccinated with SinH-3 significantly reduced bacterial burden in the total organs of ST73 and ST95 (Adjusted *P* value, *P*<0.0001, *P*<0.0001). This data is shown below, which we think is unambiguous in supporting the main contention of protection.

2. **Some awkward and incorrect language:**
1) **Line 151 “proteins were cultured in *E. coli* BL21(DE3)”. The proteins were not cultured, the *E. coli* was.**

**Author Response:** Thank you for the comments. The sentence has been corrected from “Both recombinant proteins were cultured in *E. coli* BL21(DE3)” to “Both recombinant proteins were produced by *E. coli* BL21(DE3) cultured in Lysogeny broth (LB).” (Line 176)

2) **Line 148. plasmid**

**Author Response:** Thank you. The “plasmids construction” has been corrected to “plasmid construction.” (Line 164)

3) **Line 152 “the proteins were induced”. Gene expression was induced…**

**Author Response:** Yes, thank you. The sentence has been corrected from “the proteins were induced” to “the gene expression was induced”. (Line 177)

4) **Line 157 should “PSIG” just be “PSI”?**

**Author Response:** The unit of the French pressure cell press that we used is PSIG. The PSIG refers to the gauge pressure, expressed in pounds per square inch gauge. It is a unit of pressure relative to the ambient pressure or atmospheric pressure. If measuring relative to vacuum and resolving the pressure relative to the atmosphere, 1500 PSIG could convert to 1514.696 PSI.

5) **Lines 217, 219, 225, 227 (for example) Throughout the manuscript “infection” is used instead of “injected” or “inoculated”. The animals may become infected but only sometime after injection or inoculation.**

**Author Response:** This is true, we agree. We have checked the manuscript and corrected the “infected” to the “injected” or “inoculated.” (For example, line 266, line 284, line 276, line 307, line 466, line 560, line 610)

6) **Line 391. I don’t think the SinH-based antigens were harvested by centrifugation. Likely the insoluble bacterial material was pelleted. The supernatant likely contained the antigens.**

**Author Response:** Thank you, you are correct. The SinH-antigens were in the supernatant, not in the pelleted insoluble bacterial material. The sentence has been corrected to “Bacterial cultures expressing recombinant vaccine antigens were lysed, and supernatant which contained the SinH-antigens were collected (line 445-446).”

7) **Graph in Fig 3E should read” Unvaccinated” on x-axis.**

**Author Response:** Thank you for the comment. There is no unvaccinated group in Fig. 3E. But we found the incorrect name "unvaccinated group" in Fig. 5E. The “unvaccination” in Fig. 5E has been corrected to “unvaccinated.” The same incorrect name was also found in Fig. 5C and Fig. 5D, and now the “unvaccination” has been corrected to “unvaccinated.”

8) **In Fig 5B, there is no trend in this experiment. Simply state that the differences were not significant. One cannot put such a spin on these data. Likewise for Figure 6B (SinH-123).**

**Author Response:** We don’t fully agree with this statement but have decided to make the change out of respect to the reviewer’s comment. There is absolutely a trend (a qualitative assessment), but it's certainly true that the trend is not significant (quantitatively different). The word trend can be used to connotate that there might be something happening that is different between the two groups, for it states what is actually observed (and we just need to increase N to show “true” differences). However, it certainly is also true that the difference may be normal stochastic variance. We have avoided using the word “trend” to describe such results. For example, in Fig. 6B, we removed the word “trend” in the results section and only described the data using a statistical analysis (lines 562-565). Likewise, in Fig. 7B, we also removed the word “trend” in the results section (lines 592-595).

**Reviewer #2 major comments:**

1. **The mouse models consider three rather distinctive circumstances: 1) septicemia from experimental peritonitis; 2) urinary tract infection – specifically cystitis/bladder infection; 3) gastrointestinal colonization. Each of these is associated with very different clinical contexts, immunologic responses, microenvironments, and bacterial functions. The most compelling result presented here is the vaccine mortality benefit (presented in Figure 4) observed in the septicemia model of ST73 and ST95 infections, which is corroborated by a substantial difference in organ CFUs.**

**Curiously missing from this paper are survival results for the ST131 strains presented before this in Figure 3. I see no report of mortality data for these strains in the corresponding Results section (lines 403-442). This was surprising as the SinH-3 domain from ST131 (lines 142-147) differs by 30 amino acids from the same proteins in ST73 and ST95. I see a less marked vaccine-associated CFU drop for ST131 strains compared to the non-ST131 strains.**

**Does ST131 express less antigen? Did the ST131 strains somehow evade humoral immunity to a greater degree than ST73/95?**

**Author Response:** Thank you for reviewing our study. Your comments helped improve the study and manuscript, especially the suggestion (below) of testing the vaccine for protection against ST131 mortality (and to a lesser degree the variation in the antigen amino acid composition which may affect immunogenicity).

Regarding if ST131 expresses less antigen, we do not know the answer (and this would be difficult to test in vivo during infection) but the new experiment demonstrated below indicates that a robust protective response against ST131 does occur, thereby indicating the antigen must be expressed during infection. This result also suggests that ST131 does not evade humoral immunity since that is the primary response we have observed. We expand on this discussion below in response to the reviewer’s second question.

2. **Given that the authors focused upon ST131 strains and septicemia, this is a very important result that merits more direct treatment. Please provide the data for ST131 survival so it may be directly compared to the survival data for the non-ST131 strains.**

**Author Response:** We agree, this is a good suggestion. The very first experiment performed in this study was to determine the bacterial levels of ST131 after vaccination with SinH domains; we simply never followed up on a mortality study after that initial result with ST131, which means we had to execute a new study. In this case, we designed a vaccine and challenge study using ST131 strain JJ2050, a strain with broad AMR that is also virulent in the murine model of bacteremia.

In this experiment, we decided to add two additional vaccination/challenge cohorts which also helped us address a few control suggestions listed elsewhere in this review (so this description is similar to the one already put forth above). These control groups are an Alum-only group (30 μl/mouse, N=8) and an LPS-only group (3 EU/mouse, N=8). There were two test cohorts, similar to most of the studies in this report. They are: SinH-3 (50 μg antigen mixed with 30 μl Alum adjuvant, N=12) and SinH-123 (50μg antigen mixed with 30 μl Alum adjuvant, N=12). Six-week-old female BALB/cJ mice were given three subcutaneous injections of Alum-only, LPS-only or the SinH-antigens on days 0, 14, and 28 as throughout. Strain JJ2050 was injected intraperitoneally (5 × 10^7^ CFU) on day 42. Murine survival was followed with time and moribund animals were euthanized/necropsied to determine bacterial levels in the kidneys, spleen, and liver. The organs were homogenized, and the JJ2050 bacterial load in the infected organs was quantified by the determination of CFU. The survival rate curve was determined using the Gehan-Breslow-Wilcoxon comparison. Significant was determined by the Kruskal-Wallis analysis of variance (ANOVA) with Dunn’s multiple comparisons correction. Symbols represent data of individual mice. One star (*) *P* < 0.05, two stars (**) *P* < 0.01, three stars (***) *P* < 0.001, four stars (****) *P* < 0.0001.

The vaccination and challenge schema used in this experiment is shown below.

Results: Mice vaccinated with an LPS-only, or Alum-only controls all died within 1 day-post-infection. In contrast, animals immunized with either of the SinH antigens showed a survival rate of 33% after 10-days (Adjusted *P* value, SinH-3, *P*=0.0037; SinH-123, *P*=0.0090). [Alum group or LPS group: 8/8 mice died in 1 d.p.i; SinH-3 group: 4/12 mice died in 1 d.p.i, 4/8 mice died in 2 d.p.i, and 4/4 mice survived until the end of 10 days; SinH-123 group: 5/12 mice died in 1 d.p.i, 3/7 mice died in 2 d.p.i, and 4/4 mice survived until the end of 10 days]. (**Data shown below**)

The bacterial levels in SinH vaccinated animals showed results consistent with the survival data. Combining the counts from all organs, compared to the mice vaccinated with LPS-only, the mice vaccinated with SinH-3 significantly reduced the bacterial burden of JJ2050 in organs after 2 d.p.i (Adjusted *P* value, *P*=0.0248) and after 10 d.p.i (Adjusted *P* value, *P*<0.0001). Also, compared to the mice vaccinated with alum-only, the mice vaccinated with SinH-3 significantly reduced the bacterial burden of JJ2050 in organs after 10 d.p.i (Adjusted *P* value, *P*<0.0001). Compared to the mice that received LPS-only or alum-only, SinH-3 vaccinated mice had a 4-log reduction or 3.8-log reduction in the median level of JJ2050 strain after 10 d.p.i.

Likewise, compared to the mice vaccinated with LPS-only or alum-only, the SinH-123 vaccinated mice also had significantly reduced bacterial burdens in organs after 2 d.p.i (Adjusted *P* value, *P*=0.0023, *P*=0.0281) and 10 d.p.i (Adjusted *P* value, *P*<0.0001, *P*<0.0001). In comparison to the mice that received LPS-only or alum-only, SinH-123 vaccinated mice had a 6.6-log reduction or 6.4-log reduction in the median level of JJ2050 strain after 10 d.p.i. **(data shown below).**

When taken together, these results indicate that vaccination with the SinH antigens could protect against a lethal challenge of ST131 *E. coli*. Both SinH antigens increased at least 33% survival rate and reduced bacterial burden by several logs (4-6 log) in major organs.

We have added this new data to the revised manuscript.

**Reviewer #2 other comments:**

1. **Assuming equivalent antigen expression (not examined in this manuscript), among the three models tested (sepsis, UTI, intestinal colonization), I would expect the humoral immune response to subcutaneous vaccination to have the greatest impact upon septicemia because of circulating antibodies, as is found here. Since these invasive infections are what kill patients, I regard this as an important, if not the most important, outcome of the current study.**

**Author Response:** Thank you, we agree, and this is why we were excited to report this finding to *PloS Pathogens*.

2. **I am concerned that the current manuscript understates this distinction (lines 32-35, for example). The survival data in Fig 4 provides the clearest example of protection, while the meaning of the relatively narrow CFU differences associated with other organisms and contexts is more difficult to discern. While perhaps an imperfect analogy, the concept that a subcutaneous vaccine could confer greater protection against an invasive disease than to a pathogen confined to the mucosa has become highly familiar in the age of COVID vaccines.**

**Author Response:** Thank you. With the new data presented above (ST131 experiment suggested by the reviewer), we believe this point is highlighted to a greater degree in the revised manuscript. We specifically state now that these antigens are protective against at least three very virulent and problematic sequence types (131, 73, and 95).

3. **Unmentioned in this manuscript is the subject of sinH transcription and expression as a modulating influence on the results. What is known about this? Are there any clues from the sinH promoter region?**

**Author Response:** This is an interesting direction to pursue. Neither we nor any others that we know have examined SinH transcription. As we tried to highlight in the manuscript and addressed above in Reviewer 1’s comments, there has been almost no study of the biology of this protein. The primary objective of this study and the funded work, in general, was to discover and evaluate new ExPEC antigens for vaccine development. Because of this objective, examining the regulation of SinH was somewhat beyond the scope of this work (and probably outside the expertise of our group).

However, to become a bit more educated on this topic and try to provide some clarity for us and the reviewer’s point, we did examine the 100 bps upstream of *sinH* (putative promoter region) to look for signatures that may give clues into transcription. In the figure below, we show the consensus, sequence, and identity histogram for the promoter regions of *sinH* from phylogroups A, B2, D, E, F, and G. As one can see from this figure, the 100-bps proceeding *sinH* are generally highly conserved, but there is some phylogroup specific variation. In addition, the ST131 group has some variation, but it is overwhelmingly very similar to non-ST131s.

Figure. The promoter region of sinH in different phylogroups. MAFFT was used to align the 100 base pairs upstream of the sinH start codon for each phylogroup. The consensus sequence, sequence logo, and identity histogram are shown. (A) promoter sequence for each phylogroup. Note that the B2 phylogroup has three separate diagrams: All B2 strains (ST131 + non-ST131 strains), just ST131 strains, and just non-ST131 strains to highlight the variability between these two groups. (B) A similar alignment representation is shown for all strains, where one diagram shows all strains while the other shows all non-ST131 strains.

4. **Line 552: I agree with the authors’ suggestion that Fc-mediated effects that are not active at mucosal surfaces or intestinal/bladder lumen is a potential explanation for the diminished effect of vaccination on colonization**.

**Can the authors comment on the likelihood that antibodies that bind SinH would affect SinH-mediated bacterial functions?**

**Author Response:** This is the topic of the next study. The reviewer is getting at the mechanism of protection which we will move on to as a follow-up manuscript after this one. That being said, we can speculate about a potential neutralization of the SinH function.

Because the SinH protein is the invasin-like autotransporter adhesin protein, the Fab (antigen-binding fragment) fragment of the SinH-antigens specific antibodies might have the function of neutralization of SinH epithelial cell interactions (adherence blocking), invasion of ExPEC into cells (which may prevent dissemination), or both. A second idea might be that SinH’s surface-bound antibodies might promote opsonophagocytosis. This immunological clearance function might be the reason for an increase in survival rate, and a significant reduction of the bacterial loads of the SinH-antigens vaccinated mice in different *E. coli* sequence types (ST) 0-48 hours. Likewise, the Fab fragment might also thereby restrict the dissemination of bacteria via the bloodstream and prevent the translocation in the mice. Other possibilities include antibody-dependent cell-mediated cytotoxicity (ADCC), antibody-dependent cellular phagocytosis (ADCP), and/or complement-dependent cytotoxicity (CDC). Future studies will focus on ruling in or out such mechanisms.

5. **Has anybody examined virulence of a SinH knockout?**

**Author Response:** Yes, to some degree. As far as we can tell there is only one study on the function of this protein. The paper “Loss of an Intimin-Like Protein Encoded on a Uropathogenic *E. coli* Pathogenicity Island Reduces Inflammation and Affects Interactions with the Urothelium (DOI: https://doi.org/10.1128/iai.00275-21)” expound on some in vitro experiments of *sinH* knockout strains. In this paper, the authors generated the ΔSinH mutant CFT073 *E. coli* strain. They demonstrated that ΔSinH mutants did not result in measurable effects on growth, biofilm formation, or motility in vitro. Furthermore, the authors performed a series of assays to quantify interactions with both bladder and kidney cell lines in vitro. Unexpectedly, the results showed that compared to WT CFT073, a ΔSinH mutant displayed a significant increase in bladder cells in vitro. However, after investigating the ability of UPEC to internalize into T24 bladder cells, the ΔSinH mutant resulted in a significant loss of intracellular bacteria and cell death, which indicates that SinH ultimately contributes to the invasion of cultured bladder cells after adherence.

In addition, the authors conducted a co-challenge experiment by transurethrally inoculating with 10^8^ CFU containing equal numbers of the ΔSinH mutant and the WT strain. The results showed that the ΔSinH mutant displayed a significant colonization defect during infection in the urine, bladder, and kidney.

In general, and by combining with our results above, it seems sinH is important for bladder colonization and necessary for bacterial infection, thereby indicating that a vaccine harboring it as a component might be protective against these effects. Indeed, we observed some of this in our UTI model.

6. **On a related note, Line 556: urinary IgG didn’t “demonstrate more effective protection” in this study, rather was associated with greater protection.**

**Author Response:** Thank you for the comment. The sentence has been corrected from “more effective protection” to “a greater level of protection” (line 674).

**Reviewer #3 major comments:**

1. **L298-335. The authors claim the sequence of SinH from ST131 is significantly divergent compared to the SinH sequence from ST73 and ST95. What is the level of divergence? My quick analysis indicated the SinH protein sequence identity between reference ST131 strains compared to reference ST73 and ST95 strains is ~90% identity.**

**Author Response:** First, we thank the reviewer for the time put into critiquing this study. The comments helped improve the overall manuscript. “Significantly divergent” was too strong an adjective to use in this context. The modified manuscript now states the exact identity and similarity between a consensus sequence from the mentioned sequence types. This has been corrected (lines 374-376).

Now the sentence has been read “SinH encoded by ST131 *E. coli* appear to have somewhat diverged compared to other ExPEC causing-sequence types of the B2 phylogroup such as ST73 (88.6% identical, 93.2% similar), ST95 (88.8% identical, 92.9% similar), and ST127 (90.6% identical, 95.2% similar), especially in domain 3.”

**Following on from this, I found Fig 1 very difficult to interpret. In panel A, black boxes indicate ‘no results’ – what does this mean?**

**Author Response:** Our sincerest apology for the confusion of the first figure. It is our obligation to make the figures interpretable for maximal evaluation of the data. The confusion largely stems from not clearly explaining what the black boxes represented. They represent “No results,” which indicates that there is no sequence type (ST) present for the listed pathotype. For example, for UPECs (pathotype), there are only seven sequence types (ST10, ST5082, ST73, ST95, ST127, ST131, and ST648 – scan the columns after looking under “UPEC”), but only five of them actually contain a *sinH* homolog (their color indicative of the percent difference from the reference *sinH* gene used in this analysis). The white box indicates there is a sequence type for that pathotype, but that sequence type does not contain a *sinH* gene homolog. This has been more clearly stated in the revised figure legend for the black boxes, which now reads:

“the black boxes indicate there is no sequence type (ST) present for the listed pathotype whereas white boxes indicate there is a sequence type but it does not contain a *sinH* homolog.”

We also improved the phylogenetic tree in this figure by reordering it so that the B2 SinH were grouped together rather than divided by the phylogroup A/D/G SinH clade. This was a purely cosmetic change made by swapping sister branches.

**I could not resolve any information from panel B and panel C as the text was too small.**

**Author Response:** We have removed the small text from figures 1B and 1C. This text indicated the accession number of the strains, but this is of limited value to the figure as the important point is the distribution of sequence types and phylogroups.

2. **The SinH-3 and SinH-123 recombinant proteins were not pure - the SDS-PAGE analysis for both protein preparations shows multiple bands, including a significant band at ~25 kDa (see Fig 2F).** **The authors need to determine if these bands are breakdown products or impurities.**

**Author Response:** This is a good point and one we followed up on in the revised manuscript to include a gel of the actual antigen preparations used in the study **(Data shown below)**.

In the new gel, we see there are three strong protein bands, one at about 40 kDa, one at about 70 kDa, and one at about 26 kDa. We identified these bands by mass spectrometry. For the GST-SinH-3, the protein band around 40 kDa is the GST-SinH-3. Likewise, for the GST-SinH-123, the protein band around 70 kDa is the GST-SinH-123. The cover maps of the MS results are shown below.

Because the plasmid we used for both GST-SinH-3 and GST-SinH-123 was pGEX-2TK, which generates an N-terminal GST-tagged protein upon expression, and both proteins are expected to be GST-tagged proteins. The protein band around 26 kDa under both GST-SinH-3 and GST-SinH-123 bands was determined to be free GST, likely a cleavage product because of the proteolytic recognition sequence between GST and SinH. The spectra for this are shown below.

3. **They should also determine the purity of the sample using mass spectrometry and demonstrate there is no LPS contamination. These experiments are critical, as the authors need to demonstrate that the reduced cfu counts resulting from vaccination are due to an antibody response against SinH rather than against other ExPEC proteins (or LPS) co-purified in the procedure.**

**Author Response:** This is also a strong point. It should be noted that the above mass spectrometry results did not yield any masses consistent with LPS contamination. However, we performed an additional set of experiments to address the reviewer’s point more directly. The details are presented below.

The first experiment we performed was to determine if there was LPS in the preparations. We used the Pierce™ Chromogenic Endotoxin Quant Kit (Thermo Scientific, Waltham, MS) to determine the LPS concentration in the diluted SinH-3 and SinH-123 proteins (1:100 dilution) **(Data shown below)**.

According to the data, the standard curve for a concentration calculation adheres to **y=1.139x+0.3876** (where y represents the Avg. OD (405 nm) and x represents the LPS concentration in EU/ml). Hence, the average LPS concentration of GST-SinH-3 (1:100 dilution) was 1.15 EU/ml, and the average LPS concentration of GST-SinH-123 (1:100 dilution) was 1 EU/ml. With some basic back calculations, the original LPS concentration of GST-SinH-3 was determined to be 115 EU/ml, and that of GST-SinH-123 to be 100 EU/ml.

In the vaccination step of our previous experiments, the concentration of purified recombinant proteins (both GST-SinH-3 and GST-SinH-123) was kept at around 1.5 mg/ml to 2 mg/ml. Therefore, when we vaccinated mice by injecting 25 μl to 30 μl antigen solution into each mouse, we also injected around 3 EU LPS into the mouse each time (a total of three times). This is not by any measure a high level of LPS (in fact, its below what is allowed for contamination in any human-approved therapeutics, even adjusted to the weight of an average person).

To definitely rule out that LPS could be responsible for the strong protection observed herein, we decided to perform an entirely new vaccination experiment to determine the protection afforded by SinH vs LPS. Thus, we used a cohort of mice given the exact amount of LPS as determined above, designated here the “LPS-only” group (3 EU/mouse, N=8). The SinH-3 (50 μg antigen mixed with 30 μl Alum adjuvant, N=12) and the SinH-123 (50 μg antigen mixed with 30μl Alum adjuvant, N=12) were the experimental groups. Six-week-old female BALB/cJ mice were given three subcutaneous injections of LPS-only and SinH-antigens on days 0, 14, and 28.

ExPEC sequence type 131 (ST131) strains JJ2050 were grown under the indicated conditions as described in the manuscript. Mice were injected intraperitoneally by 50μl of the JJ2050 strains suspension (5 × 10^7^ CFU) on day 42. Mice were monitored twice a day for 10 days. Murine survival was followed with time, and moribund animals were euthanized/necropsied to determine bacterial levels in the kidneys, spleen, and liver. The organs were homogenized, and the JJ2050 bacterial load in the infected organs was quantified by the determination of CFU. The survival rate curve was determined using the Gehan-Breslow-Wilcoxon comparison. Significant was determined by the Kruskal-Wallis analysis of variance (ANOVA) with Dunn’s multiple comparisons correction. Symbols represent data of individual mice. One star (*) *P* < 0.05, two stars (**) *P* < 0.01, three stars (***) *P* < 0.001, four stars (****) *P* < 0.0001.

The vaccination schema used in this experiment is shown below.

All the LPS-only vaccinated control group mice died one day-post-infection (0% survival). In contrast, the mice immunized with the SinH antigens showed a 33% survival rate in the 10-day challenge period (Adjusted *P* value, SinH-3, *P*=0.0037; SinH-123, *P*=0.0090). (LPS group: 8/8 mice died in 1 d.p.i; SinH-3 group: 4/12 mice died in 1 d.p.i, 4/8 mice died in 2 d.p.i, and 4/4 mice survived until the end of 10 days; SinH-123 group: 5/12 mice died in 1 d.p.i, 3/7 mice died in 2 d.p.i, and 4/4 mice survived until the end of 10 days) **(data shown below)**.

The bacterial levels in SinH vaccinated animals showed results consistent with the survival data. Combining the counts from all organs, compared to the LPS-only group mice, the mice vaccinated with SinH-3 significantly reduced bacterial burden in organs of JJ2050 after 2 d.p.i and 10 d.p.i. (Adjusted *P* value, 2 d.p.i, *P*=0.0300; 10 d.p.i, *P*<0.0001). Likewise, compared to the LPS-only group mice, the SinH-123 vaccinated mice also significantly reduced the bacterial burden in organs of JJ2050 after 2 d.p.i and 10 d.p.i. (Adjusted *P* value, 2 d.p.i, *P*=0.0026, 10 d.p.i, *P*<0.0001). Compared to the mice that received LPS-only, SinH-3 vaccinated mice had a 4.03-log reduction, and SinH-123 vaccinated mice had a 6.61-log reduction in the median level of JJ2050 strain after 10 d.p.i **(data shown below).**

These results indicate that the LPS-only vaccinated group did not provide any protection against ST131 *E. coli* JJ2050 mortality or reduction in bacterial levels, whereas both SinH antigens increased at least 33% survival rate with substantial reductions (4-6 log) in bacterial levels 10 d.p.i. Taken together, these data suggest that LPS is not responsible for the observed protection of the SinH antigens.

4. **It would be relevant to employ the 5-day IP infection protocol to assess vaccine protection and survival (the critical readout) with the ST131 strains. Please comment.**

**Author Response:** Thank you, this information has now been included in the manuscript.

5. **Is the data showing protection against colonization of the bladder by UTI89 from a single experiment? If so, this experiment should be repeated to demonstrate reproducibility.**

**Author Response:** Thank you, it was from a single cohort of mice vaccinated and challenged in staggard. We are not sure this qualifies as a true biological replicate, so we decided to repeat this entire experiment again. We purchased 18 BALB/CJ mice (Unvaccinated group, N=8; SinH-3 vaccinated group, N=10, female, age 6 weeks) and performed the vaccination, challenge, and assessment as described in the original manuscript. Experimental female BALB/CJ mice were immunized subcutaneously (50μg SinH-3, mixture 2:1 with Alum adjuvant) on days 0, 14, and 28. Mice were inoculated transurethrally on day 42 with 1x10^8^ CFU of UTI89 strain. We allowed the infection to last for 72 hours, bladders were homogenized, and the UTI89 bacterial load in the infected organs was quantified **(data shown below).**

According to the data, SinH-3 vaccinated mice had a 69-fold reduction in the median levels of UTI89 colonization in the bladder compared to the unvaccinated mice, with an adjusted P value of p=0.0960) just short of statistical significance. When combined with the previous data, the sum of all studies was statistically significant, and by a wide margin (p = 0.0051). This indicates vaccination with SinH-3 is protective against UTI89 colonization in the bladder **(data shown below)**.

6. **Likewise, is the gut colonization data from a single experiment?**

**Author Response:** No, the results demonstrated are two completely separate gut colonization experiments performed at different times, the only difference being one was without chemotherapy, and the other was with chemotherapy. Chemotherapy is meant to create an immunosuppressive environment. In both cases, the SinH-3 vaccinated animals had reduced colonization. (One might consider these three and four separate experiments if one also considers the sinH-1,2,3 immunizations).

7. **I have concerns about the statistical methods used to evaluate the significant difference in cfu counts in different experiments. Data in Fig 3 and Fig 5 were evaluated using a two-tailed Mann-Whitney test or Kruskal-Wallis test (which one is not specified in the figure legend), while data in Fig 4 and Fig 6 were evaluated using a two-tailed Mann-Whitney test. Please explain. Given there are multiple comparisons, I would have expected these analyses would be performed using a test such as the Kruskal-Wallis analysis of variance (ANOVA) with Dunn’s multiple comparisons correction. Please justify the choice of statistical method.**

**Author Response:** Thank you for your comments. Both the Mann-Whitney-Wilcoxon test and the Kruskal-Wallis analysis of variance (one-way ANOVA on ranks) with Dunn’s multiple comparisons correction (in short, the Kruskal-Wallis test) are nonparametric methods designed to detect whether 2 or more samples come from the same distribution or to test whether medians between comparison groups are different. The major difference between the Mann-Whitney-Wilcoxon test and the Kruskal-Wallis test is that the latter can accommodate more than two groups (of three or more independent groups).

We understand the reviewer’s concern. Hence, to be more consistent between experiments, we used the Kruskal-Wallis analysis of variance (ANOVA) with Dunn’s multiple comparisons correction to re-analyze all the data in the three models. All the p-values in the manuscript and figures were revised to adjusted p-values by Dunn’s multiple comparisons correction, and the corresponding description has also been revised in the manuscript (line 475, line 494-496, line 517, line 522-524, line 528-529, line 531, line 546-547, line 551, line 565-567, line 574, line 579, line 582, line 594, line 615-618).

**Reviewer #3 other comments:**

1. **L410-411. Please define the genetically distinct clonal groups for JJ1886, JJ2050 and JJ2547.**

**Author Response:** We thank the reviewer for bringing this to our attention. All three of these strains are ST131s that belong to the C2, or H30Rx, clade. This sentence has been updated for clarity.

The sentence has been corrected from “These strains were chosen because they are genetically distinct clonal groups.” to “These strains were chosen because they represent a diverse range of strains from the genetically distinct and epidemic clade—clade C2, or H30Rx—of the ST131 clonal group [98].” (Line 467-469)

2. **Were the mice sick at 24 hours post infection with the ST131 strains? The data for the ST73 and ST95 strains would indicate that many of the mice may have died within this time (i.e., compare with survival data in Fig 4.**

**Author Response:** Thank you for the comments. Yes, many mice were sick 24 hours post-infection with the ST131 strains. To get a more accurate representation of this, we redesigned a 10-day ST131 JJ2050 survival curve supplemental experiment (details listed above).

In this experiment, we showed that in the control group, 8/8 mice died 1 d.p.i. In the SinH-3 group, 4/12 mice died 1 d.p.i, 4/8 mice died in 2 d.p.i, and 4/4 mice survived until the end of 10 days. In the SinH-123 group, 5/12 mice died 1 d.p.i, 3/7 mice died 2 d.p.i, and 4/4 mice survived until the end of 10 days.

3. **Please refer to strains by their strain name rather than ST. As noted by the authors, there is considerable variation at the ExPEC genome level, and thus while the range of strains examined is to be commended, single strains are not representative of an entire ST.**

**Author Response:** Thank you, it’s a good point. To make the manuscript more accurate in this regard, we added the specific strains' names after the sequence type (ST) rather than ST only. We described the ST73 and ST95 strains we used in the previous experiments (in the materials and methods, line 158-161) and the strains we used in the ST73-mixture and the ST95-mixture more accurately (line 308-310, line 542-543).

4. **In the discussion, it is stated that ‘from the perspective of epidemiology, a vaccine against ExPEC would be expected to be effective against many of the 50 million incident cases of sepsis and 11 million sepsis-related deaths worldwide and would contribute to the reduction of the recurrence rate of complicated UTI.**

**The authors should comment on a how a vaccine for E. coli would be used. How will they identify the high-risk target group? Surely it would not be administered to everyone.**

**Author Response:** Thank you. A few comments on this.

1. A recently published sepsis incidence and mortality study paper in Lancet mentioned (Rudd et al., 2020) that among all ages, both sexes, and all underlying causes, an estimated 41.5 million incident sepsis cases in 2017 occurred in countries with a low, low-middle or middle SDI (Socio-demographic Index (SDI) is a composite indicator of development status strongly correlated with health outcomes). Also, the highest age-standardized sepsis-related mortality occurred in areas with the lowest SDI. An estimated 8·2 million sepsis-related deaths in 2017 occurred in countries with low, low-middle, or middle SDIs. According to the statistical data above, we believe the people of low, low-middle, or middle SDI countries and districts would benefit from an *E. coli* vaccine to reduce sepsis-related deaths and incidence.
2. Urinary tract infections (UTIs) are the most common outpatient infections, with a lifetime incidence of 50-60% in adult women (Medina et al., 2019). Even though our SinH-based vaccine didn’t show prominent strong protection against UPEC colonization on the bladder, we believe that with the optimization (dosage, vaccination method, or adjuvant), a SinH protein-subunit vaccine would protect against UPEC colonization. For this reason, women, especially those over 50 years old, would be a target population.
3. As mentioned in the manuscript, solid-organ transplantation (SOT) has become the preferred strategy for a large variety of end-stage organ conditions. However, bacterial infections, particularly bloodstream infections (BSIs), remain the most common and life-threatening complications in SOT recipients. More specifically, because of the frequent urinary origin of BSIs in kidney transplant recipients, Gram-negative bacteria are usually identified as the primary pathogens responsible for BSI. For example, pathogenic *Escherichia coli* was responsible for up to 30% of episodes of BSI (Kritikos et al., 2016). For this reason, our *E. coli* vaccine could be an important preventive strategy to reduce the incidence of post-transplant BSIs and control the spread of MDR organisms in the transplantation.
4. From the age-related sepsis incidence perspective, overall, sepsis incidence peaked in early childhood, with a second peak in incidence among older adults (Rudd et al., 2020). For example, in 2017, there were an estimated 20.3 million incident sepsis cases and 2·9 million deaths related to sepsis worldwide among children younger than 5 years. Hence, we suggest that our *E. coli* vaccine could be used to vaccinate children younger than 5 years and older adults (over 50 years) to reduce the sepsis incidence and death rate among this population.
5. **Although SinH is immunogenic, the results actually show it is not a protective antigen. For example, at 24h bacteria were still present in the blood of all mice (Fig 3).**

**Even more concerning from a clinical perspective, the challenge strain was present in the spleen and kidney in high numbers in immunized mice (albeit at lower cfu numbers than the control), indicating a lack of protection in these tissues. The authors should comment.**

**Author Response:** Thank you. The very first experiment performed in this study was to determine the bacterial levels of ST131 after vaccination with SinH domains; we simply never followed up on a mortality study after that initial result, which means we had to execute a new study. In this case, we designed a vaccine and challenge study using ST131 strain JJ2050, a strain with broad AMR that is also virulent in the murine model of bacteremia. The results of 10-day experiments indicated the protection of the SinH-antigens against pathogenic *E. coli*.

The first experiment is the ST131 *E. coli* JJ2050 strain survival curve experiment. The data and the details are listed above. In short, Six-week-old female BALB/cJ mice were given three subcutaneous injections of SinH-antigens on days 0, 14, and 28 (50 μg antigen mixed with 30μl Alum adjuvant). Mice were injected intraperitoneally with 50μl of the JJ2050 strains suspension (5 × 10^7^ CFU) on day 42. Mice were monitored twice a day for 10 days. Murine survival was followed with time, and moribund animals were euthanized/necropsied to determine bacterial levels in the kidneys, spleen, and liver. The organs were homogenized, and the JJ2050 bacterial load in the infected organs was quantified by the determination of CFU. Significant was determined by the Kruskal-Wallis analysis of variance (ANOVA) with Dunn’s multiple comparisons correction. Symbols represent data of individual mice. One star (*) *P* < 0.05, two stars (**) *P* < 0.01, three stars (***) *P* < 0.001, four stars (****) *P* < 0.0001.

According to the results, after being challenged with *E. coli* ST131 JJ2050 strain, combining the counts from all organs, the 10 d.p.i (N=4) SinH-antigen vaccinated mice showed a significantly lower level of bacterial loads compared to the 2 d.p.i (N=8) of SinH-antigen vaccinated mice (Adjusted *P* value, SinH-3, *P*=0.0006; SinH-123, *P*<0.0001). These results indicate the total number of JJ2050 burden in the SinH-vaccinated group across different organs consistently declined, which means the SinH-antigen vaccine-specific antibodies show some level of prophylactic protection against dissemination.

Likewise, we also used the same model to test the level of protection of SinH-3 against the UTI89 (ST95) *E. coli* strain in a 10-day experiment. In this model, female BALB/CJ mice were immunized subcutaneously three times as described in the previous model, and mice were intraperitonally injected on day 42 with 1 × 10^8^ CFU of UTI89 strains **(data shown below).**

According to the data, combining the counts from all organs, we showed the total number of UTI89 colonization of the SinH-3 vaccinated group across different organs consistently declined over time, with no bacteria found in most of the organs in the 10 d.p.i group mice.

Taken together, especially in the context of the several mortality curves, this data clearly shows that the SinH antigens are highly protective against pathogenic and virulent *E. coli*. Keep in mind that in the real world, the patient would never be exposed to the doses we are exposing these mice to (in some cases, many times the lethal dose 50 and millions to hundreds of millions of cells at one time). We fully expect that if these results translate to humans, there would be sterilizing immunity in many cases since such doses would never be encountered in the clinical or natural world.

6. **Do the authors envisage SinH would be used as part of a multivalent vaccine? Some discussion is warranted.**

**Author Response:** This is a good question. A single protein subunit vaccine is not the final step of our long-term goal on this project. We have identified other candidates for this project which have not yet been tested but have been discussed in a recent manuscript from our group (Clark, Infection and Immunity, 2021).
