# Supplementary material for: Broad protective vaccination against systemic Escherichia coli with autotransporter antigens
Source: PLoS Pathog. 2023 Feb 17;19(2):e1011082. doi: 10.1371/journal.ppat.1011082 (PMC9937491; doi:10.1371/journal.ppat.1011082)
Supplement: Attachment — Submitted filename: Responses to Reviewers - Final Version.docx [file ppat.1011082.s003.docx]

**Anthony William Maresso, Ph.D.**

Joseph Melnick Endowed Chair and Professor

One Baylor Plaza, BCMC 203A

MS: BCM280

HOUSTON, TEXAS 77030-3411

832-446-7284

[maresso@bcm.edu](mailto:maresso@bcm.edu)

[TAILΦR Labs](https://www.bcm.edu/research/labs-and-centers/research-centers/tailor)

Twitter [@BCMTailorLabs](https://twitter.com/BCMTailorLabs?s=20)

December 2^nd^, 2022

Kimberly Kline

Christoph Tang

Kasturi Haldar

Michael Malim

Editors

*PLOS Pathogens*

**RE:** Submission of a Revised manuscript titled, “*Broad Protective Vaccination against Systemic Escherichia coli with Autotransporter Antigens”*.

Dear Managing Editor,

Thank you for serving as the Managing Editor for our manuscript. This revised manuscript addresses a few remaining editorial changes asked for by reviewers 2 and 3. Reviewer 1 had no further comments.

These changes are:

1. Clarification of the interpretation regarding colonization.
2. Clarification of the interpretation of a previously published results concerning ST131.
3. Clarification of CFU counts from moribund animals.
4. Textual correction concerning beta lactamases.

A detailed description of these changes can be found in the document labeled “Response to Reviewers.”

We hope the revised manuscript is now suitable for publication in *PloS Pathogens* and thank you again for review.

Anthony William Maresso, Ph.D.

Melnick Endowed Chair and Professor

Baylor College of Medicine

**RESPONSE TO REVIEWERS**

The authors wish to thank the reviewers for their time in reviewing this study, which helped improve the manuscript. A point-by-point response to the reviewer’s comments are indicated below, as are any changes that were made to the revised manuscript (indicated by line numbers).

**Reviewer #1 (Comments for the Author):** **The authors have bent over backwards to answer all critiques of all three reviewers. The manuscript is greatly improved. None.**

**Reviewer #2 (Comments for the Author):**

1) **Lines 583-598: The data in this section is explicitly characterized by the subheading as demonstrating that GI tract colonization is reduced by immunization (this is also a conclusion in Discussion lines 632-4), yet none of the results for this are significant and the effect sizes are small. The data as presented does not support the stated conclusion. Immunization has a non-significant, minimal effect on GI colonization by ExPEC strains**

**Author Response:** We concede the reviewer’s point and have now changed this heading and the indicated lines to reflect this fact.

1. For the subheading, we changed the wording to “SinH-based vaccination led to a minimal reduction in ExPEC colonization in healthy mice that was not statistically significant” (lines 598-599).
2. For the conclusion (v) in the Discussion, we noticed that using the word “effective” is a stretch in our interpretation of the data. Hence, we rewrote the conclusion (v) in the Discussion section to reflect this: “that whereas immunization with SinH-based antigens did lead to a reduction in colonization compared to the control in both healthy and immunocompromised GI tract mice models, this was not statistically significant.” (lines 647 – 650)
2) **Lines 520-526: given the high mortality of the infection, are the bacterial levels measured here only from survivors? This should be stated more clearly in this subsection.**

**Would not be surprised to see lower CFU in survivors and agree it is worth presenting these data as explicitly confirmatory of the survival data. A summary of the data in this subsection in which mortality and CFU analyses are related would be useful for readers.**

**Author Response:** No, the bacterial levels are not only from survivors. The CFU counts come from animals that were either moribund according to our disease index score (and thus were euthanized with organs homogenized and plated for bacterial levels – LPS (black bars), Alum (blue bars), and sinH-vaccinated (2 d.p.i. brown bars) only cohort in Fig. 4C) or from surviving animals from SinH-vaccinated cohorts (not determined to be moribund in fact fully healthy/recovered) at 10 d.p.i. (red bars).

In the modified manuscript we now indicate in the Results section whether the mice were moribund and thus euthanized versus surviving and healthy and euthanized at the end of the experiment. (lines 531 – 539, 540 – 545)

3) **Consider restatements of the following:**

- **Line 104-106: I don’t think this was the authors’ interest but the suggestion that a beta lactamase has fueled fluoroquinolone resistance sounds like a misunderstanding of beta-lactamase function, which would not be expected to directly affect fluoroquinolone susceptibility**.

**Author Response:** Good point. We agree the sentence is misleading. It has now been changed to “which has fueled the emergence of resistance ExPEC strains globally [9,28].” (lines 105)

- **Line 106-107: “…unlike the other ExPEC sequence type, ST131 has more extensive genome content and an expanded number of virulence genes…”.**

**This is a misleading statement, as the results in the cited review come from a comparative genomic study to a specific set of antibiotic-resistant ExPEC sequence types.**

**Unlike that comparison, the present study compares ST131 to the more common ExPEC ST73, 95, and 127. It may be that the identity and combination of virulence genes, not the number of them, is determinative for ST131 pathogenicity and transmission. This is not a critical point to the manuscript but is a detail worth clarifying.**

**Author Response:** Thank you, we generally agree with the reviewer’s assessment of that paper. For a more precise description of the ST131 in the introduction, we have made the changes listed below:

(1) Like the reviewer mentioned above, ST131 has some particular combination of virulence genes, which showed unique virulence profiles. Hence, we cited the original paper (DOI: <https://doi.org/10.1128/mBio.01596-17>, in the reference of the manuscript is [100]) and added a sentence about the unique virulence profiles of ST131 in the introduction (lines 105 – 109).

The sentence added was “Additionally, unlike the other non-ST131 ESBL-producing E. coli lineages (ST38, ST405, and ST648), ST131 genomes showed unique virulence profiles. For example, the presence of several serine proteases, autotransporters, and UPEC-specific virulence genes were identified exclusively in ST131 isolates [100].” (lines 105 – 109).

(2) The original text of the review paper that we cited in the manuscript stated that (verbatim) (DOI: <https://doi.org/10.1128/CMR.00135-18>) “*ST131 possessed a greater total number of virulence genes, while the non-ST131 isolates shared similar, albeit lower, numbers of virulence genes, but the composition of genes varied (199).*” For a clearer description, we have rewritten the sentence in the manuscript (lines 109 – 110) and cited both the review and the original papers.

The new sentence on this point reads “Furthermore, ST131 has an expanded number of virulence genes in contrast to the non-ST131 isolates, which shared similar but varied and lower numbers of virulence genes [1,100].” (lines 109 – 110)

We hope this is a more accurate representation of that work. It seems that both are true – that the total number of virulence genes as well as their combination might drive ST131 virulence phenotypes.

- **Is the seventh conclusion in lines 636-637 the same as the first conclusion in lines 627-628? This seems like the primary conclusion stated once more.**

**Author Response:** Thank you. We suppose the conclusions are similar, but we meant them to be different for they reference two separate experiments with different outcomes.

1. For the first conclusion (i), we indicate that both two antigens, SinH-3 and SinH-123, showed protection against ST131 (the strain that causes the most infection globally), with increased the survival rate in a bacteremia model.
2. Meanwhile, seventh conclusion (vii), we emphasize that mice vaccinated with SinH-3 demonstrate a significant increase in survival rate after infection with ExPEC ST73 and 95.

So, there are two differences in the stated conclusions. The first difference is the sequence type (131 versus 73 and 95). The second difference is in antigens (SinH-3 and SinH-123 for conclusion 1 and only SinH-3 for conclusion seven).

- **Lines 645-646: “a vaccine against ExPEC… would contribute to the reduction of the recurrence rate of complicated UTI”. Singling out “complicated UTI”, which is a clinical subcategory of UTI, seems odd here. Why not just say it would be effective against UTI or recurrent UTI?**

**Author Response:** You are correct and very astute. We appreciate that you pointed out that having a complicated UTI is odd in this context. The sentence has now been changed from “recurrence rate of the complicated UTI” to “UTI and recurrent UTI.” (line 661)

**Reviewer #3 (Comments for the Author):** **This revised manuscript is significantly improved. I have the following additional comments.**

1. **L106-7. This is incorrect. ST131 does not have more extensive genome content and an expanded number of virulence genes compared to other ExPEC.**

**Author Response:** Thank you, we generally agree with the reviewer’s assessment of that paper. For a more precise description of the ST131 in the introduction, we have made the changes listed below:

(1) Like the reviewer mentioned above, ST131 has some particular combination of virulence genes, which showed unique virulence profiles. Hence, we cited the original paper (DOI: <https://doi.org/10.1128/mBio.01596-17>, in the reference of the manuscript is [100]) and added a sentence about the unique virulence profiles of ST131 in the introduction (lines 105 – 109).

The sentence added was “Additionally, unlike the other non-ST131 ESBL-producing E. coli lineages (ST38, ST405, and ST648), ST131 genomes showed unique virulence profiles. For example, the presence of several serine proteases, autotransporters, and UPEC-specific virulence genes were identified exclusively in ST131 isolates [100].” (lines 105 – 109).

(2) Thank you for pointing out that ST131 does not have more extensive genome content. We have deleted this part of the statement.
(3) The original text of the review paper that we cited in the manuscript stated that (verbatim) (DOI: <https://doi.org/10.1128/CMR.00135-18>) “*ST131 possessed a greater total number of virulence genes, while the non-ST131 isolates shared similar, albeit lower, numbers of virulence genes, but the composition of genes varied (199).*” For a clearer description, we have rewritten the sentence in the manuscript (lines 109 – 110) and cited both the review and the original papers.

The new sentence on this point reads “Furthermore, ST131 has an expanded number of virulence genes in contrast to the non-ST131 isolates, which shared similar but varied and lower numbers of virulence genes [1,100].” (lines 109 – 110)

We hope this is a more accurate representation of that work.

2. **L115-116. I am not aware that O antigen vaccines exhibit weak immunogenicity.**

**Author Response:** You are correct and thank you for bringing this to our attention. The “weak” here means that compared with the whole LPS, the O-antigen exhibit “weak” immunogenicity. Some papers have shown that the O-antigen conjugate vaccine showed good immunogenicity and tolerability in animal studies. The major problem of the limited further development of the O-antigen *E. coli* vaccine is the “antigenic heterogeneity of the surface polysaccharides of the *E. coli*.”

The sentence has been corrected from “weak immunogenicity of the O-antigen vaccines” to “antigenic heterogeneity of the surface polysaccharide of the *E. coli.”* (lines 120-121)

3. **L585-587. I don’t understand this statement. Please reword for clarity. ‘In addition, in fecal *E. coli* strains carrying ExPEC-associated virulence factors, up to 93.5% of fecal isolates classified as InPEC additionally carried ExPEC virulence factors.’**

**Author Response:** Thank you for your comment. This cited paper demonstrates that the “*E. coli* genome is not a static entity, and hybrid strains might be associated with several distinct virulence factors.” They showed a surprisingly high number of traditionally classified InPEC strains that carried ExPEC virulence-associated genes in the feces samples of the GI tract, which might cause the disease outside of the GI tract rather than a traditional InPEC. This sentence in the manuscript attempted to add a rationale for the GI tract model and lead to the next sentence, the importance of reduction of the colonization of ExPEC or *E. coli* strains to carry ExPEC-associated virulence factors.

For clarity, we have rewritten the sentence from “In addition, in fecal *E. coli* strains carrying ExPEC-associated virulence factors, up to 93.5% of fecal isolates classified as InPEC additionally carried ExPEC virulence factors” to “In addition, a recent study showed up to 93.5% of traditionally classified InPEC fecal isolates additionally carried ExPEC virulence factors, which might cause the infection outside of the GI tract.” (lines 600 – 602)

4. **P583-598. The gut colonization experiments are confusing. In the methods this is written as one experiment with 2 parts. However, in the results it is written as 2 independent experiments, and this is also depicted in Fig 7A and 7D. Please clarify.**

**Author Response:** We must have been confusing in our description. As mentioned in the previous response, the results demonstrated in that study are two separate gut colonization experiments. The reason for writing the material and methods together is to avoid excess wordiness in this section.

To correct this, we have now revised the methods to reflect this fact (lines 295 – 303).

5. **Why were cfu’s only determined from feces at one timepoint? If more data is available, this should be shown, even if the differences are not significant between the groups.**

**Author Response:** Thank you. We only have the feces sample from one time point of both GI tract model experiments (24 hours post-infection or 24 hours post-chemotherapy). So, we do not have the data at other time points. We would have shown that data had we had it.

The reason for only collecting one feces sample is that we wanted to be consistent with the experimental procedure from the previous bacteremia and UTI model. For example, we collected the bladder 24 hours post-infection after transurethral injection in the UTI model; collected different organs 24 hours post-infection after intraperitoneal injection in the bacteremia model. Hence, we only collected feces samples once 24 hours post-infection after gavage in the GI tract healthy mice model and once in the GI tract immunosuppressed model 24 hours after the chemotherapy.

6. **L632-4. This statement is not consistent with the data. Please correct. ‘(v) that immunization with SinH-based antigens are effective at reducing the colonization of ExPEC ST131 in the GI tract of healthy or immunocompromised mice’**

**Author Response:** Thank you. Reviewer #2 also states this and so we have decided to change the heading and conclusions as indicated below.

1. For the subheading, we changed the wording to “SinH-based vaccination led to a minimal reduction in ExPEC colonization in healthy mice that was not statistically significant” (lines 598-599).
2. For the conclusion (v) in the Discussion, we noticed that using the word “effective” is a stretch in our interpretation of the data. Hence, we rewrote the conclusion (v) in the Discussion section to reflect this: “that whereas immunization with SinH-based antigens did lead to a reduction in colonization compared to the control in both healthy and immunocompromised GI tract mice models, this was not statistically significant.” (lines 647 – 650)
